# Phenotyping of Eggplant Wild Relatives and Interspecific Hybrids with Conventional and Phenomics Descriptors Provides Insight for Their Potential Utilization in Breeding

**DOI:** 10.3389/fpls.2016.00677

**Published:** 2016-05-19

**Authors:** Prashant Kaushik, Jaime Prohens, Santiago Vilanova, Pietro Gramazio, Mariola Plazas

**Affiliations:** Instituto de Conservación y Mejora de la Agrodiversidad Valenciana, Universitat Politècnica de ValènciaValencia, Spain

**Keywords:** descriptors, genepools, intespecific hybrids, introgression breeding, phenomics, *Solanum melongena*, Tomato Analyzer

## Abstract

Eggplant (*Solanum melongena*) is related to a large number of wild species that are a source of variation for breeding programmes, in particular for traits related to adaptation to climate change. However, wild species remain largely unexploited for eggplant breeding. Detailed phenotypic characterization of wild species and their hybrids with eggplant may allow identifying promising wild species and information on the genetic control and heterosis of relevant traits. We characterizated six eggplant accessions, 21 accessions of 12 wild species (the only primary genepool species *S. insanum* and 11 secondary genepool species) and 45 interspecific hybrids of eggplant with wild species (18 with *S. insanum* and 27 with secondary genepool species) using 27 conventional morphological descriptors and 20 fruit morphometric descriptors obtained with the phenomics tool Tomato Analyzer. Significant differences were observed among cultivated, wild and interspecific hybrid groups for 18 conventional and 18 Tomato Analyzer descriptors, with hybrids generally having intermediate values. Wild species were generally more variable than cultivated accessions and interspecific hybrids displayed intermediate ranges of variation and coefficient of variation (CV) values, except for fruit shape traits in which the latter were the most variable. The multivariate principal components analysis (PCA) reveals a clear separation of wild species and cultivated accessions. Interspecific hybrids with *S. insanum* plotted closer to cultivated eggplant, while hybrids with secondary genepool species generally clustered together with wild species. Many differences were observed among wild species for traits of agronomic interest, which allowed identifying species of greatest potential interest for eggplant breeding. Heterosis values were positive for most vigor-related traits, while for fruit size values were close to zero for hybrids with *S. incanum* and highly negative for hybrids with secondary genepool species. Our results allowed the identification of potentially interesting wild species and interspecific hybrids for introgression breeding in eggplant. This is an important step for broadening the genetic base of eggplant and for breeding for adaptation to climate change in this crop.

## Introduction

Eggplant (*Solanum melongena* L.) is an important vegetable in tropical and subtropical regions across the world, where it is a source of dietary fiber, micronutrients and bioactive compounds (Mennella et al., 2010; Niño-Medina et al., 2014; San José et al., 2014). At present eggplant is the sixth most important vegetable after tomato, watermelon, onion, cabbage, and cucumber and the most important *Solanum* crop native to the Old World (FAO, 2016). At the global level, it has been one of the crops with the greatest increase in production in the last years, with total production rising by 59% in a decade, from 31.0·10^6^ t in 2004 to 49.3·10^6^ t in 2013 (FAO, 2016).

The narrow genetic base of eggplant, probably a consequence of a genetic bottleneck during its domestication in Southeast Asia (Meyer et al., 2012), is a limitation to obtain major breeding advances. This limited genetic diversity contrasts with the large morphological and genetic variation present in the eggplant wild relatives (Meyer et al., 2012; Vorontsova et al., 2013; Vorontsova and Knapp, in press). Phylogenetically, eggplant is a member of the so-called “spiny solanums” group (*Solanum* subgenus *Leptostemonum*), which contains many wild species from the Old World, most of them from Africa (Vorontsova et al., 2013; Vorontsova and Knapp, in press). These wild species could represent a source of variation for developing a new generation of eggplant cultivars with dramatically improved yield and quality, as well as for addressing the challenges posed by adaptation to the climate change. In this respect, resistance and tolerance to several major diseases and pests is found among wild eggplant relatives (Daunay and Hazra, 2012; Rotino et al., 2014) and they can also be found in a wide range of environmental conditions, including desertic and semi-desertic areas, environments with extreme temperatures (Knapp et al., 2013; Vorontsova and Knapp, in press). Some eggplant wild relatives are known to possess high levels of chlorogenic acid and other bioactive compounds of interest for human health (Mennella et al., 2010; Meyer et al., 2015). However, with a few exceptions (Rotino et al., 2014; Liu et al., 2015), eggplant breeders have largely neglected the potential of wild species for eggplant breeding, and contrarily to other crops like tomato (Díez and Nuez, 2008), wild relatives have not made a relevant contribution to the development of new eggplant cultivars.

Eggplant can be crossed with a large number of wild relatives (Daunay and Hazra, 2012; Rotino et al., 2014; Plazas et al., 2016). The closest wild relative of eggplant is *S. insanum* (Knapp et al., 2013; Vorontsova et al., 2013), which is naturally distributed in Southeast Asia, Madagascar and Mauritius (Knapp et al., 2013; Vorontsova and Knapp, in press), where it is frequently found as a weed (Mutegi et al., 2015). *Solanum insanum* is considered as the wild ancestor of eggplant and is the only species in the primary genepool of cultivated eggplant (Syfert et al., 2016). Hybrids of eggplant with *S. insanum* are easily obtained; fruits from interspecific hybridization have many seeds, which have high germination rates, and the hybrid plants are fully fertile (Davidar et al., 2015; Plazas et al., 2016). Interspecific hybrids have also been obtained with many wild species from the secondary genepool (Daunay and Hazra, 2012; Rotino et al., 2014; Plazas et al., 2016), which includes some 50 African and Southeast Asian species (Vorontsova et al., 2013; Syfert et al., 2016). The degree of success of interspecific sexual hybridization between eggplant and secondary genepool species, as well as the hybrid fertility is variable depending on the species involved and the direction of the cross (Plazas et al., 2016).

The characterization of wild species and interspecific hybrids for traits of interest for breeders is a fundamental step for the efficient utilization of crop wild relatives in breeding. Combined data on the cultivated and wild species and their interspecific hybrids, not only allows identifying sources of variation and materials of potential interest, but also provides information on the inheritance of some traits present in the wild species, as has been demonstrated in crosses between *S. incanum* and eggplant (Prohens et al., 2013). Also, characterization of these materials for vigor traits may allow identification of materials potentially useful as rootstocks. In this respect, highly vigorous eggplant of wild relatives and interspecific hybrids are increasingly used for eggplant grafting, as they induce precocity and higher yield and many of them are tolerant to biotic and abiotic stresses (Gisbert et al., 2011; Daunay and Hazra, 2012). In the case of eggplant wild relatives there are a number of studies on their taxonomic and phylogenetic relationships (Vorontsova et al., 2013; Vorontsova and Knapp, in press), of resistance or tolerance to diseases and pests (Bubici and Cirulli, 2008; Daunay and Hazra, 2012; Naegele et al., 2014). However, to our knowledge there are no comprehensive studies on the morphological and agronomic traits of interest in a set of wild species of the primary and secondary genepools of eggplant and their interspecific hybrids with cultivated eggplant.

Several characterization studies in eggplant with standardized morphological and agronomic descriptors developed by the European Eggplant Genetic Resources Network (EGGNET; van der Weerden and Barendse, 2007) and the International Board for Plant Genetic Resources (IBPGR, 1990) have revealed that are suited for providing a useful morphological and agronomic characterization for eggplant breeders (Prohens et al., 2005; Muñoz-Falcón et al., 2009; Boyaci et al., 2015). EGGNET and IBPGR descriptors have been successfully used for evaluating segregating generations of interspecific crosses between eggplant and related species (Prohens et al., 2012, 2013). In addition to conventional morphological descriptors fruit phenomics data provide eggplant breeders with relevant information for evaluating the variation of the fruit morphology. In this respect, the phenomics tool Tomato Analyzer (Rodríguez et al., 2010) has revealed as useful for the detailed morphometric analysis of fruit size and shape of eggplant and related materials (Prohens et al., 2012; Hurtado et al., 2013).

Here we characterize cultivated eggplant, wild relatives from the primary and secondary genepools and interspecific hybrids between cultivated eggplant and wild relatives using conventional and Tomato Analyzer descriptors. Apart from providing a characterization of the three types of materials studied and their differences, we aim to evaluate the interest for breeding of different wild relatives using characterization data of the wild relatives and of their interspecific hybrids with eggplant. The information obtained may also provide clues on the interest of wild species and hybrids as potential rootstocks for eggplant.

## Materials and methods

### Plant material

The plant material included six accessions of cultivated eggplant (*S. melongena*), 21 accessions of a total of 12 wild species, and 45 interspecific hybrids between the eggplant accessions and seven of the wild species (Table 1). The eggplant accessions include materials from both the Occidental (Ivory Coast) and Oriental (Sri Lanka) cultivated genepools (Vilanova et al., 2012; Cericola et al., 2013). Among the wild relatives, three accessions belong to the primary genepool (GP1) *S. insanum*, and 18 accessions to secondary genepool (GP2) species, namely *S. anguivi* (*n* = 2), *S. campylacanthum* (*n* = 3), *S. dasyphyllum* (*n* = 1), *S. incanum* (*n* = 1), *S. lichtensteinii* (*n* = 2), *S. lidii* (*n* = 2), *S. linnaeanum* (*n* = 2), *S. pyracanthos* (*n* = 1), *S. tomentosum* (*n* = 1), *S. vespertilio* (*n* = 2), and *S. violaceum* (*n* = 1). All the accessions are deposited at the germplasm bank of the Universitat Politècnica de València (València, Spain). The 45 interspecific hybrids were obtained after reciprocal crossings between cultivated eggplant and wild relatives (Plazas et al., 2016) resulting in 18 hybrids between eggplant and primary genepool species and 27 hybrids between eggplant and secondary genepool species (Table 1). Five plants per accession or interspecific hybrid were grown under open field conditions during the summer season of 2015 at the Universitat Politècnica de València (Valencia, Spain; GPS coordinates of the plot: 39° 28′ 55″ N, 0° 22′ 11″ W; altitude 7 m a.s.l.). Plants were spaced 1.2 m between rows and 1.0 m within the row and distributed according to a completely randomized design. Drip irrigation was applied and 80 g plant^−1^ of a 10N–2.2P–24.9K plus micronutrients fertilizer (Hakaphos Naranja; Compo Agricultura, Barcelona, Spain) was applied during the whole cultivation period through the irrigation system. Plants were trained with bamboo canes and pruned when needed. Weeds were removed manually and no phytosanitary treatments were needed.

**Table 1:** **Accessions of cultivated eggplant (*Solanum melongena*) and wild relatives of the primary and secondary genepools, and interspecific hybrids between cultivated eggplant and wild relatives used for the morphological and phenomics characterization**.

**Species**	**Accession**	**Germplasm collection code**	**Country of origin**	**Interspecific hybrids with cultivated eggplant accessions**					
				**MEL1**	**MEL2**	**MEL3**	**MEL4**	**MEL5**	**MEL6**
**CULTIVATED EGGPLANT**									
*S. melongena*	MEL1	BBS-118/B	Ivory Coast						
	MEL2	BBS-146	Ivory Coast						
	MEL3	BBS-175	Ivory Coast						
	MEL4	07145	Sri Lanka						
	MEL5	8104	Sri Lanka						
	MEL6	Ampara	Sri Lanka						
**WILD PRIMARY GENEPOOL (GP1)**									
*S. insanum*	INS1	SLKINS-1	Sri Lanka	MEL1 × INS1	MEL2 × INS1	MEL3 × INS1	MEL4 × INS1	INS1 × MEL5	MEL6 × INS1
	INS2	SLKINS-1	Sri Lanka	MEL1 × INS2	MEL2 × INS2	MEL3 × INS2	MEL4 × INS2	MEL5 × INS2	MEL6 × INS2
	INS3	MM498	Japan	INS3 × MEL1	INS3 × MEL2	INS3 × MEL3	INS3 × MEL4	MEL5 × INS3	INS3 × MEL6
**WILD SECONDARY GENEPOOL (GP2)**									
*S. anguivi*	ANG1	BBS119	Ivory Coast		MEL2 × ANG1	MEL3 × ANG1	MEL4 × ANG1	MEL5 × ANG1	
	ANG2	BBS125/B	Ivory Coast	MEL1 × ANG2	MEL2 × ANG2	ANG2 × MEL3	ANG2 × MEL4	MEL5 × ANG2	ANG2 × MEL6
*S. campylacanthum*	CAM5	MM680	Tanzania						
	CAM6	MM700	Kenya						
	CAM8	MM1426	Tanzania						
*S. dasyphyllum*	DAS1	MM1153	Uganda	MEL1 × DAS1	MEL2 × DAS1	MEL3 × DAS1		MEL5 × DAS1	
*S. incanum*	INC1	MM664	Israel	INC1 × MEL1		MEL3 × INC1		MEL5 × INC1	MEL6 × INC1
*S. lichtensteinii*	LIC1	MM674	South Africa	MEL1 × LIC1				MEL5 × LIC1	MEL6 × LIC1
	LIC2	MM677	Iran	MEL1 × LIC2		MEL3 × LIC2	MEL4 × LIC2		
*S. lidii*	LID1	4788	Spain						
	LID2	MM1005	Spain						
*S. linnaeanum*	LIN1	JPT0028	Spain						LIN1 × MEL6
	LIN3	MM195	Tunisia						
*S. pyracanthos*	PYR1	SOLN-66	Unknown						
*S. tomentosum*	TOM1	MM992	South Africa		MEL2 × TOM1	TOM1 × MEL3			
*S. vespertilio*	VES1	4601A	Spain						
	VES2	BGV-3218	Spain						
*S. violaceum*	VIO1	SLKVIL-1	Sri Lanka						

*For the interspecific hybrids, the first and second parentals included in the hybrid code correspond to the female and male, respectively*.

### Characterization

All plants were characterized using 27 conventional morphological descriptors based on EGGNET (van der Weerden and Barendse, 2007) and IBPGR (IBPGR, 1990) descriptors (Table 2). These morphological descriptors describe different traits of the whole plant (4), leaf (7), inflorescence and flower (7) and fruit (9) and in general display limited GxE interaction (IBPGR, 1990). Except for descriptors concerning the whole plant (e.g., plant growth habit), for which one measurement was taken per plant (i.e., one measurement per replicate), five measurements were taken from each individual plant in order to obtain individual plant averages for the conventional morphological descriptors (i.e., five measurements per replicate). Using a similar approach, five fruits per plant (replicate), collected at the commercially ripe stage (i.e., physiologically immature) for cultivated eggplant and at a similar physiological stage (when they had attained full size but was not physiologically mature) in the case of wild species and interspecific hybrids, were cut opened longitudinally and scanned using an HP Scanjet G4010 photo scanner (Hewlett Packard, Palo Alto, CA, USA) at a resolution of 300 dpi. Scanned images were subjected to fruit morphometric analysis with the fruit shape phenomics tool Tomato Analyzer version 4 software (Rodríguez et al., 2010). A total of 20 fruit morphometric descriptors were recorded using this tool (Table 2).

**Table 2:** **Descriptors used for phenotyping**.

**Descriptors**	**Units/Scale/Description**
**CONVENTIONAL MORPHOLOGICAL DESCRIPTORS**	
Plant growth habit	3 = Upright; 7 = Prostrate
Plant height	cm
Stem diameter	mm
Shoot tip anthocyanin intensity	0 = Absent; 9 = Very strong
Leaf blade lobing	1 = Very weak (none); 9 = Very Strong
Leaf prickles (upper surface)	0 = None; 0 = Very many (>20)
Leaf surface shape	1 = Flat; 9 = Very convex or bullate
Leaf blade tip angle	1 ≤ 15°; 9 ≥ 160°
Leaf pedicel length	cm
Leaf blade length	cm
Leaf blade width	cm
Number of flowers per inflorescence	−
Corolla color	1 = Greenish white; 9 = Bluish violet
Corolla diameter	mm
Number of flower prickles (calyx)	0 = None; 9 = Very many (>20)
Number of sepals	−
Number of petals	−
Number of stamens	−
Fruit pedicel length	mm
Fruit pedicel diameter	mm
Fruit length/Breadth ratio	1 = Broader than long; 9 = Several times as long as broad
Fruit cross section	1 = Circular, no grooves; 9 = Very irregular
Fruit apex shape	3 = Protruded; 7 = Depressed
Fruit weight	g
Fruit flesh density	1 = Very loose; 9 = Very dense
Fruit calyx length (relative)	1 = Very short (< 10%); 9 = Very long (>75%)
Fruit calyx prickles	0 = None; 9 = Very many (>30)
**TOMATO ANALYZER PHENOMICS FRUIT MORPHOMETRIC DESCRIPTORS**	
Perimeter	cm
Area (A)	cm^2^
Width mid-height	The width measured at 1/2 of the fruit's height (cm).
Maximum width	The maximum horizontal distance of the fruit (cm).
Height mid-width	The height measured at 1/2 of the fruit's width (cm).
Maximum height	The maximum vertical distance of the fruit (cm).
Curved height	The height measured along a curved line through the fruit (cm).
Fruit shape index external I	The ratio of maximum height to maximum width.
Fruit shape index external II	The ratio of height mid-width to width mid-height.
Curved fruit shape index	The ratio of curved height to the width of the fruit at mid-curved-height.
Proximal fruit blockiness	The ratio of the width at the upper blockiness position to width mid-height.
Distal fruit blockiness	The ratio of the width at the lower blockiness position to width mid-height.
Fruit shape triangle	The ratio of the width at the upper blockiness position to the width at the lower blockiness position.
Ellipsoid	The ratio of the error resulting from a best-fit ellipse to the area of the fruit. Smaller values indicate that the fruit is more ellipsoid.
Circular	The ratio of the error resulting from a best-fit circle to the area of the fruit. Smaller values indicate that the fruit is more circular.
Rectangular	The ratio of the area of the rectangle bounding the fruit to the area of the rectangle bounded by the fruit.
Shoulder height	The ratio of the average height of the shoulder points above the proximal end point to maximum height.
Obovoid	Calculated according to the formula provided in the Tomato Analyzer manual (Rodríguez et al., 2010). The higher the value, the greater is the area of the fruit below mid height.
Ovoid	Calculated according to the formula provided in the Tomato Analyzer manual (Rodríguez et al., 2010). The higher the value, the greater is the area of the fruit above mid height.
Fruit shape index internal	The ratio of the internal ellipse's height to its width.

*The list displays conventional morphological descriptors based on EGGNET (van der Weerden and Barendse, 2007) and IBPGR (1990) descriptors list and phenomics fruit morphometric descriptors based on Tomato Analyzer software (Rodríguez et al., 2010) used for the characterization of accessions of cultivated eggplant (S. melongena; n = 6); wild relatives (n = 21) and interspecific hybrids between cultivated eggplant and wild relatives (n = 45)*.

### Data analyses

For each trait, the mean, range and coefficient of variation (CV, %) were calculated using average accession or hybrid values of cultivated eggplant (*n* = 6), wild relatives (*n* = 21) and interspecific hybrids (*n* = 45). Means of each accession or hybrid were subjected to analyses of variance (ANOVA) to detect differences among the three groups considered. Significance of differences among group means was evaluated using the Student-Newman-Keuls multiple range test at *P* = 0.05. Heterosis over mid parent (H; %) for the traits of greater agronomic importance was studied in the interspecific hybrids using formula H = 100 × ((F1 − MP)/MP), where F_1_ = hybrid mean, and MP = mean of the parents. Values of H above 100% indicate that the hybrid is superior to the highest parent, and therefore present positive heterosis over the highest parent. Principal components analyses (PCA) were performed using pairwise Euclidean distances among accession or hybrid means for standardized characterization data. All the statistical analyses were performed using the Statgraphics Centurion XVI software (StatPoint Technologies, Warrenton, VA, USA).

## Results

### Differences between eggplant, wild relatives and interspecific hybrids

Significant differences (*P* < 0.05) were found among average values for the groups constituted by cultivated eggplant, wild relatives and interspecific hybrids for 18 out of the 27 conventional descriptors (Table 3). Generally, wild species and interspecific hybrids had larger plant size, greater leaf prickliness, more flowers per inflorescence, and less elongated fruits than the cultivated species. The cultivated species and interspecific hybrids had more anthocyanin pigmentation, larger leaf size, and greater number of flower parts than the wild species. Flower, fruit pedicel and fruit size had the greater average values in the cultivated species, while the smaller ones were for the wild species, with the interspecific hybrids having intermediate values. The three groups overlap for all conventional descriptors except for Leaf Pedicel Length, Corolla Diameter, Fruit Pedicel Length, Fruit Pedicel Diameter, and Fruit Weight, in which all the accessions of the cultivated species presented higher values than any of the wild species.

**Table 3:** **Variation parameters for conventional morphological descriptors**.

**Descriptors**	**Cultivated eggplant (*n* = 6)**		**Wild relatives (*n* = 21)**		**Interspecific hybrids (*n* = 45; 42 for fruit traits)**		**F-ratio**	**Probability**
	**Mean^a^ (range)**	**CV (%)**	**Mean (range)**	**CV (%)**	**Mean (range)**	**CV (%)**		
Plant growth habit	5.33 a	15.3	4.71 a	24.3	5.00 a	0.0	2.51	0.0883
	(5.00–7.00)		(3.00–7.00)		(5.00–5.00)			
Plant height	97.1 a	16.5	124.8 b	17.5	141.9 b	19.5	9.81	0.0002
	(69.7–111.7)		(91.0–160.5)		(91.0–199.0)			
Stem diameter (mm)	22.6 a	20.3	24.3 ab	25.9	27.8 b	16.8	5.09	0.0087
	(15.3–28.0)		(12.0–34.7)		(18.3–38.3)			
Shoot tip anthocyanin intensity	3.33 b	86.3	0.57 a	211.2	2.06 ab	112.7	5.43	0.0064
	(0.00–7.00)		(0.00–3.00)		(1.00–7.00)			
Leaf blade lobing	4.33 a	23.8	4.81 ab	52.4	6.02 b	19.5	5.42	< 0.0065
	(3.00–5.00)		(1.00–9.00)		(3.00–9.00)			
Leaf prickles (upper surface)	0.11 a	244.9	3.38 b	95.6	4.45 b	66.7	6.03	0.0039
	(0.00–0.67)		(0.00–9.00)		(0.00–9.00)			
Leaf surface shape	5.67 a	28.8	5.29 a	45.2	6.33 a	30.1	1.96	0.1489
	(5.00–9.00)		(1.00–9.00)		(5.00–9.00)			
Leaf blade tip angle	5.00 a	25.3	4.48 a	32.8	4.58 a	33.1	0.29	0.7484
	(3.00–7.00)		(3.00–7.00)		(2.00–7.00)			
Leaf pedicel length (cm)	6.91 c	14.1	2.74 a	41.1	5.70 b	25.5	42.27	< 0.0001
	(5.80–8.28)		(0.63–4.61)		(2.67–9.05)			
Leaf blade length (cm)	22.0 b	7.9	13.8 a	33.7	21.0 b	19.3	23.65	< 0.0001
	(19.7–24.9)		(5.2–20.9)		(15.0–31.9)			
Leaf blade width (cm)	15.8 b	18.3	8.7 a	38.1	15.9 b	21.3	34.41	< 0.0001
	(12.5–19.5)		(3.3–18.7)		(10.8–25.7)			
Number of flowers per inflorescence	3.49 a	42.2	8.33 b	57.9	6.77 b	43.3	4.58	< 0.0135
	(1.07–5.00)		(1.00–16.10)		(2.00–14.44)			
Corolla color	5.67 a	18.2	5.57 a	37.9	6.02 a	24.1	0.58	0.5620
	(5.00–7.00)		(1.00–9.00)		(3.00–7.00)			
Corolla diameter (mm)	43.3 c	12.9	22.2 a	30.4	35.8 b	22.3	30.44	< 0.0001
	(37.2–49.9)		(7.7–30.4)		(20.4–49.9)			
Number of flower prickles (calyx)	1.83 a	100.1	3.62 a	102.5	3.64 a	85.2	0.86	0.4269
	(0.00–5.00)		(0.00–9.00)		(0.00–9.00)			
Number of sepals	5.57 b	14.1	4.81 a	8.4	5.25 b	7.2	10.69	< 0.0001
	(5.00–7.00)		(4.00–5.00)		(5.00–6.00)			
Number of petals	5.65 c	13.1	4.81 a	8.4	5.24 b	6.5	13.24	< 0.0001
	(5.00–7.00)		(4.00–5.00)		(5.00–6.00)			
Number of stamens	5.61 b	13.7	4.80 a	8.4	5.26 b	7.9	10.70	< 0.0001
	(5.00–7.00)		(4.00–5.00)		(5.00–6.22)			
Fruit pedicel length (mm)	43.8 c	15.2	17.5 a	30.1	28.2 b	44.9	16.13	< 0.0001
	(33.0–52.2)		(8.5–27.5)		(8.6–50.3)			
Fruit pedicel diameter (mm)	10.2 c	20.7	2.84 a	42.8	5.4 b	51.3	23.92	< 0.0001
	(7.0–12.2)		(1.0–5.1)		(1.0–10.3)			
Fruit length/breadth ratio	6.50 b	42.1	2.71 a	35.2	3.90 a	44.1	12.81	< 0.0001
	(1.00–8.00)		(1.00–5.00)		(1.00–7.00)			
Fruit cross section	5.67 a	18.2	6.05 a	47.5	5.45 a	41.7	0.43	0.6537
	(5.00–7.00)		(1.00–9.00)		(2.00–9.00)			
Fruit apex shape	5.33 a	36.9	5.19 a	32	5.33 a	30.9	0.05	0.9485
	(3.00–7.00)		(3.00–7.00)		(3.00–7.00)			
Fruit weight (g)	244.7 c	36.0	10.5 a	111.6	58.4 b	111.2	39.43	< 0.0001
	(94.4–354.5)		(0.4–35.7)		(0.6–224.2)			
Fruit flesh density	6.33 b	16.3	3.95 a	63.2	5.38 ab	44.4	3.60	0.0328
	(5.00–7.00)		(1.00–9.00)		(1.00–9.00)			
Fruit calyx length (relative)	2.67 a	30.6	4.62 a	57.5	4.05 a	51.5	1.85	0.1647
	(1.00–3.00)		(1.00–9.00)		(1.00–9.00)			
Fruit calyx prickles	2.00 a	54.8	3.48 a	91.3	3.19 a	95.0	0.58	0.5646
	(1.00–3.00)		(0.00–9.00)		(0.00–9.00)			

*Values represent the mean, range (between brackets), and coefficient of variation (CV; %) for the conventional morphological descriptors studied in accessions of cultivated eggplant (S. melongena; n = 6), wild relatives (n = 21) and interspecific hybrids between cultivated eggplant and wild relatives (n = 45 except for fruit traits in which n = 42) and significance of mean differences among the three groups*.

a *Means within rows separated by different letters are significantly different according to the Student-Newman-Keuls test*.

All Tomato Analyzer descriptors evaluated, except two (Rectangular and Shoulder Height) displayed significant (*P* < 0.05) differences among average values for the three groups (Table 4). For the eight Tomato Analyzer descriptors related to fruit size the cultivated eggplant presented significantly higher values than wild species, while for Ovoid it had lower values; interspecific hybrids presented intermediate values, in most cases being significantly different from both cultivated eggplant and wild species (Table 4). Cultivated eggplant had greater Distal Fruit Blockiness and Ellipsoid values than either wild species or interspecific hybrids, while wild species had higher values for Triangular than either cultivated species or interspecific hybrids. Similarly to conventional descriptors, the three groups overlap in the ranges of variation for all Tomato Analyzer descriptors except for Perimeter, Area, Height Mid-width, Maximum Height, Curved Height and Circular, in which there is no overlap between the range of variation of cultivated and wild species, with the values of the former being larger than those of the latter (Table 4).

**Table 4:** **Variation parameters for Tomato Analyzer phenomics fruit descriptors**.

	**Cultivated eggplant (*n* = 6)**		**Wild relatives (*n* = 21)**		**Interspecifi hybrids (*n* = 42)**		**F-ratio**	**Probability**
**Descriptors**	**Mean^a^ (range)**	**CV (%)**	**Mean (range)**	**CV (%)**	**Mean (range)**	**CV (%)**		
Perimeter	24.1 c	12.1	6.1 a	70.0	12.7 b	73.0	13.45	< 0.0001
	(20.2–28.0)		(2.1–16.2)		(2.4–28.2)			
Area	35.4 c	20.5	3.8 a	129.5	15.4 b	109.0	13.47	< 0.0001
	(24.4–42.2)		(0.3–17.2)		(0.4–46.9)			
Width mid-height	5.21 b	22.8	1.87 a	68.2	3.08 a	66.0	8.80	0.0004
	(4.01–7.03)		(0.63–4.93)		(0.70–7.37)			
Maximum width	5.35 b	21.7	1.88 a	68.1	3.11 a	66.1	9.22	0.0003
	(4.06–7.07)		(0.64–4.96)		(0.86–7.43)			
Height mid-width	8.17 c	18.4	1.69 a	68.9	4.09 b	77.2	15.60	< 0.0001
	(6.39–10.51)		(0.54–3.78)		(0.74–10.41)			
Maximum height	8.28 c	18.1	1.72 a	69.5	4.15 b	77.0	15.57	< 0.0001
	(6.55–10.64)		(0.55–3.90)		(0.75–10.53)			
Curved height	8.47 c	17.2	1.95 a	60.0	4.34 b	73.2	15.53	< 0.0001
	(6.93–10.81)		(0.85–4.52)		(0.99–10.62)			
Fruit shape index external I	1.64 c	30.0	0.90 a	8.4	1.22 b	22.5	21.66	< 0.0001
	(0.93–2.23)		(0.75–1.04)		(0.75–1.91)			
Fruit shape index external II	1.67 c	31.3	0.89 a	8.8	1.22 b	23.2	21.99	< 0.0001
	(0.91–2.30)		(0.74–1.03)		(0.71–1.96)			
Curved fruit shape index	1.72 c	29.9	1.13 a	13.2	1.35 b	17.7	14.32	< 0.0001
	(0.99–2.36)		(0.91–1.41)		(0.89–1.99)			
Proximal fruit blockiness	0.62 a	9.1	0.66 a	7.7	0.61 a	12.4	5.04	0.0092
	(0.55–0.71)		(0.58–0.78)		(0.36–0.74)			
Distal fruit blockiness	0.73 b	9.3	0.60 a	6.5	0.64 a	8.5	16.30	< 0.0001
	(0.65–0.77)		(0.52–0.65)		(0.52–0.75)			
Fruit shape triangle	0.86 a	16.6	1.12 b	12.6	0.97 a	16.5	9.91	0.0002
	(0.74–1.10)		(0.91–1.49)		(0.52–1.31)			
Ellipsoid	0.05 b	29.7	0.02 a	22.0	0.03 a	39.8	10.98	< 0.0001
	(0.03–0.07)		(0.01–0.03)		(0.01–0.07)			
Circular	0.16 c	52.0	0.05 a	41.7	0.09 b	54.2	14.92	< 0.0001
	(0.08–0.25)		(0.02–0.10)		(0.03–0.21)			
Rectangular	0.51 a	3.7	0.51 a	3.2	0.50 a	5.3	2.75	0.0711
	(0.49–0.54)		(0.48–0.54)		(0.41–0.53)			
shoulder height	0.01 a	56.7	0.01 a	68	0.01 a	74.1	0.23	0.7985
	(0.00–0.02)		(0.00–0.03)		(0.00–0.03)			
Obovoid	0.18 b	55.5	0.05 a	105.6	0.10 a	74.4	8.63	0.0005
	(0.04–0.29)		(0.00–0.18)		(0.00–0.31)			
Ovoid	0.03 a	160.0	0.09 b	62.6	0.05 ab	97.2	5.65	0.0054
	(0.00–0.11)		(0.00–0.21)		(0.00–0.17)			
Fruit shape index internal	1.67 c	31.4	0.90 a	8.5	1.22 b	23.3	21.71	< 0.0001
	(0.91–2.30)		(0.76–1.02)		(0.72–1.96)			

*Mean, range (between brackets), and coefficient of variation (CV; %) for the Tomato Analyzer phenomics fruit morphometric descriptors studied in accessions of cultivated eggplant (S. melongena; n = 6), wild relatives (n = 21) and interspecific hybrids between cultivated eggplant and wild relatives (n = 42) and significance of mean differences among the three groups*.

a *Means within rows separated by different letters are significantly different according to the Student-Newman-Keuls test*.

### Variation in eggplant, wild relatives, and interspecific hybrids

**Figure 1:**
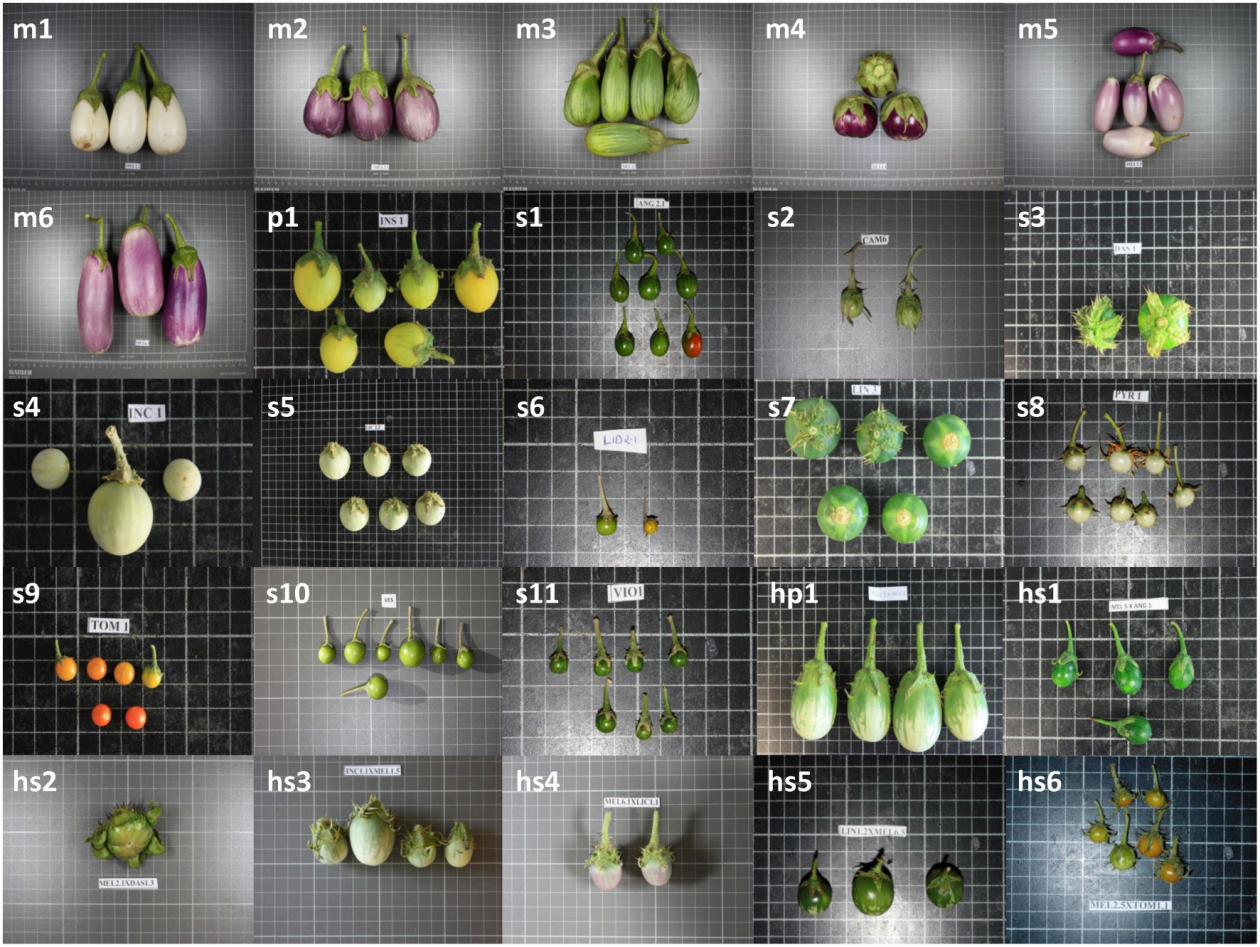
**Fruit samples of the materials used**. This include: Cultivated eggplant (*S. melongena*) accessions MEL1 (m1) to MEL6 (m6); wild species of primary genepool *S. insanum* (p1); wild species of secondary genepool *S. anguivi* (s1), *S. campylacanthum* (s2), *S. dasyphyllum* (s3), *S. incanum* (s4), *S. lichtensteinii* (s5), *S. lidii* (s6), *S. linnaeanum* (s7), *S. pyracanthos* (s8), *S. tomentosum* (s9), *S. vespertilio* (s10), and *S. violaceum* (s11); interspecific hybrids between eggplant and primary genepool species *S. insanum* (hp1); and, interspecific hybrids between eggplant and secondary genepool species S. *anguivi* (hs1), *S. dasyphyllum* (hs2), *S. incanum* (hs3), *S. lichtensteinii* (hs4), *S. linnaeanum* (hs5), and *S. tomentosum* (hs6). Fruits are not depicted at the same scale; the size of the grid cells is 1 × 1cm.

Variation for the conventional and Tomato Analyzer descriptors was found in the materials studied (Tables 3, 4; Figure 1). For most traits, more variation both in terms of range and CV was found in the wild species, compared to the cultivated eggplant accessions. For all conventional descriptors there was more variation in the wild species than in the cultivated eggplant, except for Shoot Tip Anthocyanin Intensity, the number of flower parts. Conversely, in the case of Tomato Analyzer descriptors, the range of variation was greater in wild species than in the cultivated eggplant for only six out of the 20 descriptors evaluated (Perimeter, Width Mid-height, Maximum Width, Rectangular, and Ovoid), while for the CV the wild species had a greater value than cultivated eggplant for nine of the descriptors, of which seven are related to fruit size (Table 4).

For interspecific hybrids a large range of variation was observed for many conventional descriptors, with variation parameters generally larger than those of the cultivated species and smaller than those of the wild species. In this respect, the range of variation was larger than that of the cultivated eggplant for all but nine conventional descriptors, while compared to wild species it was larger for 11 descriptors (Table 3). The coefficient of variation for conventional descriptors was also larger than in the cultivated species for all traits except nine (Plant Growth Habit, Stem Diameter, Leaf Blade Lobing, Leaf Prickles, Number of Flower Prickles, Number of Sepals, Number of Petals, Number of Stamens, and Fruit Apex Shape) and larger than that of the wild species for eight descriptors (Plant Height, Leaf Blade Tip Angle, Fruit Pedicel Length, Fruit Pedicel Diameter, Fruit Length/Breadth Ratio, Fruit Cross Section, Fruit Apex Shape, and Fruit Calyx Prickles; Table 3).

Regarding the variation for Tomato Analyzer traits, the range of variation in the interspecific hybrids was greater than those of cultivated eggplant and wild species for all traits except five in the case of cultivated eggplant, which correspond to fruit shape indexes and Circular, and only one (Ovoid) in the case of wild species (Table 4). Also, larger values were obtained in the CV for Tomato Analyzer descriptors in the interspecific hybrids compared to the cultivated species for all traits but seven. When compared to wild species the interspecific hybrids also presented higher CV for all traits, except four (Table 4).

### Multivariate analysis

The three first components of the principal components analysis made with all conventional and Tomato Analyzer descriptors accounted for 58.8% accounted of the total variation among accession means, with the first, second and third component accounting, respectively for 37.2, 12.0, and 9.5% of the total variation (Table 5). The first principal component was positively correlated to Corolla diameter, fruit size and to elongated fruit shape (Table 5). The second principal component was positively correlated to Plant Height and to obovoid fruit shape. The third principal component was positively correlated to Plant Growth Habit (i.e., prostrate habit), to multiple plant, leaf and corolla size traits, to a higher number of flower parts (sepals, petals and stamens) and to an increased prickliness in leaves, and flower and fruit calyces (Table 5).

**Table 5:** **Correlation coefficients between morphological conventional and phenomics descriptors**.

**Descriptors**	**First principal component**	**Second principal component**	**Third principal component**
Plant growth habit			0.151
Plant height (cm)		0.154	0.176
Stem diameter (mm)			0.266
Leaf blade lobing			0.258
Leaf prickles (upper surface)		−0.165	0.184
Leaf surface shape			0.236
Leaf blade length (cm)			0.291
Leaf blade width (cm)			0.306
Corolla diameter (mm)	0.184		0.153
Number of flower prickles (calyx)		−0.170	0.226
Number of sepals			0.275
Number of petals			0.267
Number of stamens			0.266
Fruit pedicel length (mm)	0.218		
Fruit pedicel diameter (mm)	0.218		
Fruit length/breadth ratio	0.191		
Fruit weight (g)	0.212		
Fruit calyx prickles		−0.190	0.253
Perimeter (cm)	0.225		
Area (cm^2^)	0.219		
Width mid-height (cm)	0.204		
Maximum width (cm)	0.206		
Height mid-width (cm)	0.231		
Maximum height (cm)	0.231		
Curved height (cm)	0.231		
Fruit shape index external I	0.209		
Fruit shape index external II	0.209		
Curved fruit shape index	0.167		
Proximal fruit blockiness		−0.371	
Distal fruit blockiness	0.163	0.204	
Fruit shape triangle		−0.349	
Circular	0.189		
Rectangular		−0.245	
Shoulder height		0.159	
Obovoid		0.328	
Ovoid		−0.312	
Fruit shape index internal	0.208		
Eigenvalue	17.50	5.65	4.48
Variance explained (%)	37.23	12.04	9.53
Cumulative variance explained (%)	37.23	49.27	58.80

*Values represent the correlation coefficients for the three first principal components in the collection of eggplant (S. melongena), wild relatives and interspecific hybrids evaluated. Only correlations with absolute values ≥0.150 have been listed*.

**Figure 2:**
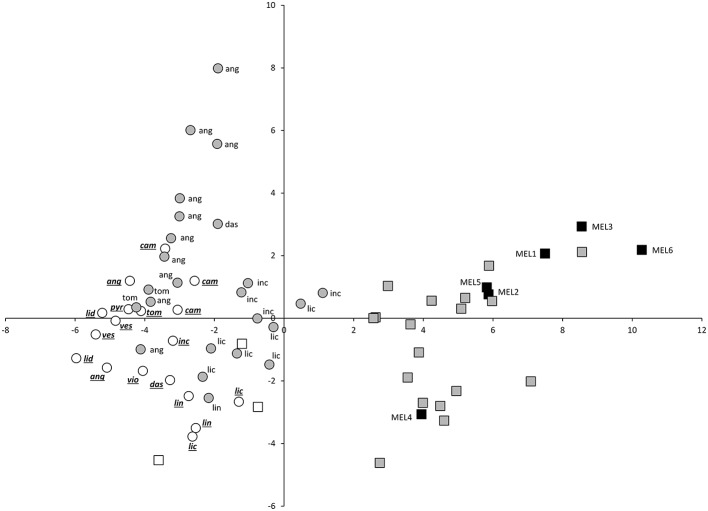
**First (X-axis) and second (Y-axis) principal components (37.2 and 12.0% of the total variation explained, respectively) scatterplot of cultivated eggplant, wild relatives and interspecific hybrids based on 27 conventional and 20 Tomato Analyzer morphological descriptors**. Cultivated eggplant (*S. melongena*) is represented by black squares and the “MEL” accession code, primary genepool species *S. insanum* by white squares, interspecific hybrids between eggplant and *S. insanum* by gray squares, secondary genepool species by white circles (with species codes in underlined italics), and interspecific hybrids between eggplant and secondary genepool species by gray circles (with wild species codes in normal font). For secondary genepool species and their hybrids with eggplant, the following codes are used: ang (*S. anguivi*), cam (*S. campylacanthum*), das (*S. dasyphyllum*), inc (*S. incanum*), lic (*S. lichtensteinii*), lid (*S. lidii*), lin (*S. linnaeanum*), pyr (*S. pyracanthos*), tom (*S. tomentosum*), ves (*S. vespertilio*), vio (*S. violaceum*).

**Figure 3:**
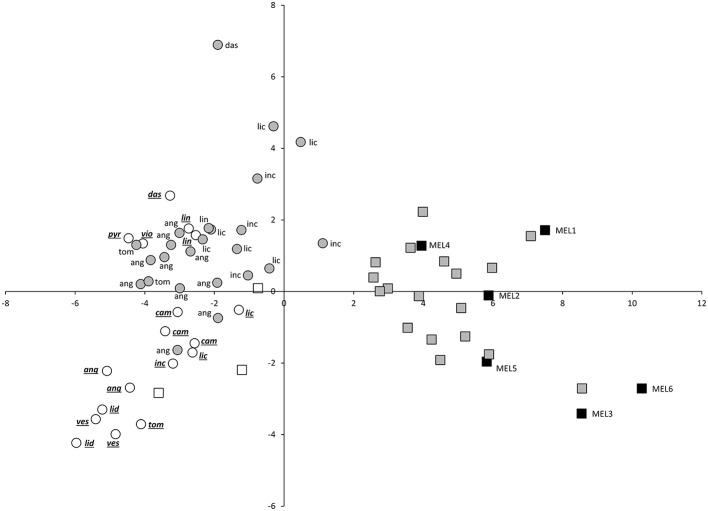
**First (X-axis) and thrid (Y-axis) principal components (37.2 and 9.5% of the total variation explained, respectively) scatterplot of cultivated eggplant, wild relatives and interspecific hybrids based on 27 conventional and 20 Tomato Analyzer morphological descriptors**. Cultivated eggplant (*S. melongena*) is represented by black squares and the “MEL” accession code, primary genepool species *S. insanum* by white squares, interspecific hybrids between eggplant and *S. insanum* by gray squares, secondary genepool species by white circles (with species codes in underlined italics), and interspecific hybrids between eggplant and secondary genepool species by gray circles (with wild species codes in normal font). For secondary genepool species and their hybrids with eggplant, the following codes are used: ang (*S. anguivi*), cam (*S. campylacanthum*), das (*S. dasyphyllum*), inc (*S. incanum*), lic (*S. lichtensteinii*), lid (*S. lidii*), lin (*S. linnaeanum*), pyr (*S. pyracanthos*), tom (*S. tomentosum*), ves (*S. vespertilio*), vio (*S. violaceum*).

The projection of eggplant, wild species and interspecific hybrids in the PCA plot reveals that although considerable diversity exists in both eggplant (black squares) and wild species (white symbols), the interspecific hybrids (gray symbols) present a more scattered distribution in the PCA plot (Figures 2, 3). Interspecific hybrids with the primary genepool species *S. insanum* plot closer to the cultivated eggplant and are intermingled with it the PCA graphs. On the contrary, interspecific hybrids with secondary genepool species plot closer to the wild species and are also intermingled with them (Figures 2, 3). The first component separates the group formed by eggplant and the interspecific hybrids with the primary genepool species *S. insanum*, which present positive values for this component, from the group formed by all the wild species and interspecific hybrids with secondary genepool species. Among the interspecific hybrids with secondary genepool species, those with *S. incanum* and *S. lichtensteinii* are the closest to eggplant in this first component (Figures 2, 3). When considering the second component all eggplant accessions but one have positive values, while interspecific hybrids with *S. insanum* are equally distributed in the positive and negative values of this second component (Figure 2). Primary genepool wild species *S. insanum* and all secondary genepool species, except *S. campylacanthum, S. pyracanthos, S. tomentosum* and one accession of each of *S. anguivi* and *S. lidii* have negative values for this second component. When considering interspecific hybrids with secondary genepool species, although they are intermingled with the wild species for this second component most of the hybrids present positive values for this second component, with the exceptions being the hybrids with *S. lichtensteinii* (four out of five), *S. linnaeanum* and one of each of the interspecific hybrids with each of the species *S. anguivi* and *S. incanum* (this latter with a value very close to 0). Amazingly, the highest values for this second component correspond to interspecific hybrids with *S. anguivi* (Figure 2). For the third component both eggplant and the interspecific hybrids with *S. insanum* are scattered and display positive or negative values (Figure 3). Most wild species accessions have negative values for this third component, except the accessions of *S. dasyphyllum, S. linnaeanum, S. pyracanthos*, and *S. violaceum*, as well as one accession of *S. incanum* (with values close to 0). The lowest values for this component are those of *S. lidii, S. vespertilio* and *S. tomentosum* (Figure 3). On the other hand all interspecific hybrids with secondary genepool species, with the exception of two interspecific hybrids with *S. anguivi*, present positive values for this third component. In this case, the highest values for the third component correspond to interspecific hybrids with *S. dasyphyllum, S. lichtensteinii*, and *S. incanum* (Figure 3).

### Traits of agronomic interest in wild species

The 12 wild species evaluated presented considerable differences for traits of agronomic interest (Table 6). For example, important differences were found for vegetative traits. For example, the tallest plants were those of *S. anguivi*, which also presented thick stems (Table 6). Important differences were also found for Leaf Blade Lobing. The greatest leaf prickliness was observed *S. dasyphyllum, S. pyracanthos*, and *S. violaceum*, while *S. anguivi* and *S. tomentosusm* did not present prickles in the leaves. The largest leaf blades were those of *S. dasyphyllum* and *S. campylacanthum*, while the smallest were those of *S. tomentosum*, with a Leaf Blade Length of 5.2 cm (Table 6). When considering flower and fruit traits, the two species with a larger number of flowers per inflorescence were *S. lidii* and *S. vespertilio*, with more than 13 flowers/inflorescence, while the smaller number was *S. insanum* (Table 6). Important differences were also observed for Corolla Color. All wild species had five petals (and sepals and stamens), except *S. lidii* and *S. vespertilio*, which had only four. The largest fruits were those of *S. incanum* and *S. lichtensteinii*, with average values above 25 g, more than 10-fold heavier than those of *S. anguivi, S. lidii, S. pyracanthos, S. tomentosum, S. vespertilio*, and *S. violaceum*. The highest calyx prickliness was observed in *S. linnaeanum, S. pyracanthos*, and *S. violaceum*, while *S. anguivi, S. lidii*, and *S. vespertilio* did not present calyx prickles (Table 6). The most elongated fruit were those of *S. incanum*, while the most flattened ones were those of *S. dasyphyllum* and *S. lidii* (Table 6).

**Table 6:** **Average (±SE) values based on accession means for selected traits in the 12 wild species (one from the primary genepool GP1, *S. insanum*; and 11 from the secondary genepool GP2) evaluated**.

**Descriptors**	**GP1**						**GP2**					
	***S. insanum***	***S. anguivi***	***S. campylacanthum***	***S. dasyphyllum***	***S. incanum***	***S. lichtensteinii***	***S. lidii***	***S. linnaeanum***	***S. pyracanthos***	***S. tomentosum***	***S. vespertilio***	***S. violaceum***
***N***	**3**	**2**	**3**	**1**	**1**	**2**	**2**	**2**	**1**	**1**	**2**	**1**
Plant height (cm)	108.7 ± 9.8	153.7 ± 6.3	150.2 ± 6.9	95.0	120.0	130.5 ± 12.5	108.6 ± 3.0	107.0 ± 0.0	141.7	104.0	115.5 ± 1.5	154.0
Stem diameter (mm)	22.8 ± 4.6	31.3 ± 2.0	21.5 ± 1.8	23.5	28.0	22.3 ± 2.4	14.0 ± 2.0	29.8 ± 0.2	34.7	19.5	28.6 ± 0.9	19.8
Leaf blade lobing	5.00 ± 0.00	2.00 ± 1.00	2.33 ± 0.67	9.00	3.00	5.00 ± 0.00	5.00 ± 0.00	9.00 ± 0.00	9.00	3.00	3.00 ± 0.00	7.00
Leaf prickles (upper surface)	3.33 ± 1.67	0.00 ± 0.00	0.67 ± 0.67	9.00	1.00	0.50 ± 0.50	6.00 ± 1.00	6.00 ± 1.00	7.00	0.00	4.00 ± 1.00	9.00
Leaf pedicel length (cm)	2.27 ± 0.51	1.98 ± 0.10	2.75 ± 0.71	1.2	2.3	2.75 ± 0.89	3.07 ± 0.33	2.99 ± 0.71	4.61	0.63	4.11 ± 0.13	3.95
Leaf blade length (cm)	8.9 ± 1.5	10.9 ± 1.6	19.1 ± 1.7	22.1	11.3	13.4 ± 2.8	14.7 ± 3.9	13.9 ± 2.4	16.9	5.2	14.3 ± 0.6	15.7
Leaf blade width (cm)	7.0 ± 1.0	7.4 ± 1.1	8.4 ± 1.4	18.7	7.8	9.3 ± 2.1	7.9 ± 3.5	9.5 ± 0.8	7.4	3.3	9.5 ± 0.7	12.9
Number of flowers per inflorescence	2.0 ± 1.0	8.2 ± 2.2	9.4 ± 1.7	10.6	9.1	5.1 ± 1.9	13.7 ± 0.7	3.0 ± 0.2	13.3	5.0	16.0 ± 0.2	10.7
Corolla color	5.67 ± 0.67	2.00 ± 1.00	7.67 ± 1.33	5.00	7.00	4.00 ± 1.00	7.00 ± 0.00	5.00 ± 0.00	9.00	3.00	5.00 ± 0.00	7.00
Number of petals	5.00 ± 0.00	5.00 ± 0.00	5.00 ± 0.00	5.00	5.00	5.00 ± 0.00	4.00 ± 0.00	5.00 ± 0.00	5.00	5.00	4.00 ± 0.00	5.00
Fruit weight (g)	26.5 ± 5.0	1.3 ± 0.5	4.6 ± 1.2	19.3	11.6	28.7 ± 6.2	0.4 ± 0.0	16.2 ± 3.3	1.0	0.5	1.2 ± 0.1	0.4
Fruit calyx prickles	3.00 ± 1.15	0.00 ± 0.00	1.67 ± 0.67	9.00	5.00	5.00 ± 0.00	0.00 ± 0.00	8.00 ± 1.00	7.00	5.00	0.00 ± 0.00	7.00
Fruit shape index external I	0.97 ± 0.39	0.95 ± 0.047	0.93 ± 0.04	0.79	1.04	0.93 ± 0.08	0.78 ± 0.04	0.94 ± 0.07	0.86	0.94	0.87 ± 0.01	0.85

*Traits were selected so that they were relevant for breeding and useful to distinguish the different wild species*.

### Heterosis in interspecific hybrids

Interspecific hybrids between eggplant and its wild relatives generally displayed positive heterosis for plant size traits, with average heterosis values of up to 90.5% for Plant height and 46.2% for Stem diameter in the hybrids of eggplant with *S. dasyphyllum* (Table 7). The only negative value observed for these traits was for Stem Diameter in the interspecific hybrid with *S. linnaeanum*. Most interspecific hybrids presented higher prickliness than their parent species, and in consequence, very high average values for heterosis for Leaf Prickles are observed, with values between 91.0% for *S. dasyphyllum* and 800.0% for *S. tomentosum*. Leaf size traits were also, in general, heterotic in the interspecific hybrids, with the exception of Leaf Pedicel Length in *S. dasyphyllum* and *S. linnaeanum*. The same phenomenon was observed for the Number of Flowers per Inflorescence, with values of up to 87.7% in the hybrids with *S. tomentosum* (Table 7). The pigmentation of the corolla (Corolla Color) also presented average positive heterosis values in the hybrids of eggplant with five out of the seven wild species, the exception being interspecific hybrids with *S. anguivi* and *S. tomentosum*. The number of flower parts, represented by the Number of Petals, displayed low absolute values for heterosis in all cases (Table 7).

**Table 7:** **Heterosis over mid parent values (%; ±SE) based on accession and interspecific hybrid means**.

**Descriptors**	***S. insanum***	***S. anguivi***	***S. dasyphyllum***	***S. incanum***	***S. lichtensteinii***	***S. linnaeanum***	***S. tomentosum***
***n***	**18**	**10**	**4/1**^a^	**4**	**6**	**1**	**2**
Plant height (cm)	16.7 ± 4.6	34.4 ± 7.1	90.5 ± 7.6	36.8 ± 11.3	38.1 ± 4.4	2.3	23.3 ± 4.2
Stem diameter (mm)	10.5 ± 4.3	10.4 ± 3.8	46.2 ± 12.3	29.1 ± 11.0	39.8 ± 10.3	−18.7	23.8 ± 3.8
Leaf prickles (upper surface)	155.1 ± 34.5	260.0 ± 173.9	91.0 ± 5.4	733.3 ± 100.0	144.4 ± 92.9	100.0	800.0 ± 800.0
Leaf pedicel length (cm)	39.7 ± 6.5	22.5 ± 7.8	−21.6 ± 1.2	19.5 ± 2.7	24.9 ± 9.2	−13.3	56.3 ± 23.9
Leaf blade length (cm)	24.9 ± 4.1	22.2 ± 5.5	34.8 ± 5.7	47.6 ± 6.6	30.6 ± 6.3	3.9	22.8 ± 1.6
Leaf blade width (cm)	27.7 ± 4.5	38.2 ± 9.5	32.9 ± 5.0	67.7 ± 9.6	41.7 ± 8.5	7.1	22.4 ± 14.0
Number of flowers per inflorescence	70.1 ± 16.0	75.9 ± 16.3	36.9 ± 13.1	21.0 ± 9.4	42.7 ± 15.7	−1.8	87.7 ± 35.5
Corolla color	15.9 ± 4.3	−2.5 ± 4.6	18.9 ± 10.4	19.2 ± 3.0	16.2 ± 4.8	7.5	−0.1 ± 8.6
Number of petals	1.3 ± 2.1	−4.8 ± 1.6	1.9 ± 5.4	−4.4 ± 2.4	−2.2 ± 3.4	−3.2	−1.0 ± 1.0
Fruit weight (g)	−5.5 ± 6.9	−98.2 ± 0.3	−60.4	−86.6 ± 2.8	−89.4 ± 1.5	−89.9	−98.6 ± 0.3
Fruit calyx prickles	32.9 ± 25.2	−100.0 ± 0.0	80.0	27.1 ± 42.4	56.9 ± 27.6	80.0	29.1 ± 104.1
Fruit shape index external I	13.7 ± 3.5	−16.7 ± 6.9	−26.4	−13.6 ± 0.8	−15.0 ± 4.3	−40.8	−27.4 ± 8.0

*Values are presented for traits of agronomic interest in the interspecific hybrids of eggplant with seven wild relatives (one from the primary genepool, S. incanum; and six from the secondary genepool)*.

a *For S. dasyphyllum data are available for four accessions for plant traits and only for one accession for fruit traits*.

Regarding Fruit Weight, considerable differences were observed between the hybrids with the primary genepool species (*S. insanum*) on one hand, and the hybrids with secondary genepool species on the other. In this respect, while the hybrids with *S. insanum* displayed small negative average heterosis (−5.5%), not significantly different from 0, in the case of secondary genepool species, the heterosis for Fruit Weight is highly negative, with values between −60.4% for hybrids with *S. dasyphyllum* to −98.6% in hybrids with *S. tomentosum* (Table 7). As occurred for Leaf Prickles, positive heterosis values, although of smaller magnitude, were observed for Fruit Calyx Prickles, with the exception of the hybrids with *S. anguivi*, which did not present prickles in the calyx, and in consequence had a heterosis value of −100%. Finally, for fruit shape, the hybrids with primary genepool species *S. insanum* presented positive heterosis, while those with secondary genepool species had negative heterosis values (Table 7).

## Discussion

Crop wild relatives are widely recognized as an invaluable genetic resource for breeding, in particular for broadening the genetic base of crops with narrow genetic diversity, and as sources of variation for traits of interest in breeding crops, including adapting them to the challenges posed by climate change (Dempewolf et al., 2014). Modern varieties of many important crops carry introgressions from wild species resulting from breeding programmes performed in the last 100 years (Hajjar and Hodgkin, 2007). One of the most outstanding examples is tomato, where modern commercial hybrids carry different combinations of 15 different introgressions from different wild species (Díez and Nuez, 2008; Sabatini et al., 2013). However, in the case of eggplant, despite being one of the most important vegetables and being intercrossable with many wild relatives, there are few reports on the use of the variation available in the wild species for eggplant breeding (Daunay and Hazra, 2012; Rotino et al., 2014; Liu et al., 2015) and no modern commercial varieties of eggplant carrying introgressions from wild species are known to us.

In our study we have evaluated six accessions of cultivated eggplant, 21 accessions of 12 wild species, and 45 interspecific hybrids of cultivated eggplant with seven wild species. This represents the largest study up to now on morphological and agronomic traits for breeding of this type of materials. As expected, many differences were found within and among cultivated eggplant, wild relatives and the interspecific hybrids for the conventional descriptors used, confirming the utility of the EGGNET (van der Weerden and Barendse, 2007) and IBPGR (1990) conventional morphological descriptors and Tomato Analyzer traits (Rodríguez et al., 2010) used for evaluating eggplant wild relatives and interspecific hybrids (Prohens et al., 2013).

Also, many differences were found for the traits studied among cultivated eggplant, wild species and interspecific hybrids. Although many of the wild species of eggplant thrive in arid and semi-arid conditions (Knapp et al., 2013; Vorontsova and Knapp, in press), when grown under the favorable conditions of cultivated environments, the wild species and their interspecific hybrids generally display a high vigor, expressed as average values for plant height and stem diameter above those of cultivated eggplant. This is of interest for developing new rootstocks, which generally require having high vigor (Gisbert et al., 2011), and opens the way to exploiting several to the wild species evaluated and interspecific hybrids as potential new rootstocks for eggplant. Another important trait of agronomic interest for which there were considerable differences among groups was prickliness, which was much greater in wild species and interspecific hybrids, confirming that alleles from the cultivated eggplant are recessive (Doganlar et al., 2002; Gramazio et al., 2014; Portis et al., 2015). The number of flowers per inflorescence was also much greater in wild species and interspecific hybrids. This trait is very important in eggplant breeding, as a reduced value of this trait results in increased fruit size uniformity (Sękara and Bieniasz, 2008). Also, fruit size and shape, which are of great relevance for breeding (Daunay and Hazra, 2012; Portis et al., 2015), also differed considerably among the three groups, with the interspecific hybrids presenting intermediate values, although on most cases they were closer to those of the wild species, indicating dominance of the genes of the latter (Doganlar et al., 2002).

The much higher variation observed in wild species and interspecific hybrids for vegetative, flower and inflorescence traits compared to cultivated eggplant was expected, as we were comparing a single species with an admixture of different wild species or hybrids, which present a much higher genetic diversity (Meyer et al., 2012; Särkinen et al., 2013; Vorontsova et al., 2013). However, for traits related to the fruit size and shape much higher variation was observed in the cultivated eggplant than in the wild species, confirming the general observation that the morphological variation in the organ for which a crop is domesticated (in this case the fruit) increases during domestication (Meyer and Purugganan, 2014). Amazingly, in the case of interspecific hybrids a larger variation was found for most fruit size and shape traits than in the cultivated eggplant. Although most interspecific hybrids were more similar to the wild species, in some cases they were intermediate, revealing that different genic control mechanisms must exist for fruit size and shape among the wild relatives of eggplant. In this respect, the multivariate analysis clearly shows that interspecific hybrids with the primary genepool species *S. insanum* are morphologically closer to the cultivated eggplant, while the hybrids with secondary genepool species present a general morphology closer to that of the wild species. These results may support the hypothesis that *S. insanum* is the wild ancestor of cultivated eggplant (Knapp et al., 2013), as domestication should be easier when genes for domestication traits from the wild species display intermediate dominance rather than full dominance.

The study of individual wild species suggests that *S. anguivi, S. campylacanthum, S. pyracanthos*, and *S. violaceum* may be of interest for increasing the vigor of cultivated eggplant or for being used as rootstocks. Also, wild eggplant species use to have undesirable traits (e.g., prickliness, small fruit size, etc.) that have to be removed during the breeding (Rotino et al., 2014). In this case, the most desirable wild species are those that are most similar to the crop for these traits. For example, the lack of prickles or very low prickliness of *S. anguivi, S. campylacanthum*, and *S. tomentosum* is a very favorable trait for breeders (Daunay and Hazra, 2012). Regarding fruit weight, the wild species with greater fruit weight should be the most interesting for breeders in order to recover fruit size in few backcross generations. In this case, *S. insanum, S. dasyphyllum*, and *S. lichtensteinii* should be the most interesting candidates if a rapid recovery of fruit size is desired. In any case, Prohens et al. (2013) showed that fruit size recovers quickly even in first backcrosses with the wild species *S. incanum*, which has an intermediate fruit size among wild species.

Although differences were observed among interspecific hybrids from different wild species, hybrids were in general vigorous, displaying heterosis for vigor traits. This phenomenon had already been described in interspecific hybrids with *S. incanum* (Gisbert et al., 2011; Prohens et al., 2013), and our results suggest that this is a common phenomenon in the hybrids between eggplant and wild relatives. Amazingly, most interspecific hybrids were highly heterotic for prickliness, with heterosis values over 100%. Prickles even appeared in interspecific hybrids with wild species that were not prickly, like *S. tomentosum*. In previous works, heterosis for prickliness had already been described in interspecific crosses in eggplant (Prohens et al., 2012; Devi et al., 2015; Plazas et al., 2016). Several studies with segregating populations of *S. linnaeanum* and *S. insanum* show that differences in prickliness between cultivated eggplant and wild relatives is under the control of a few QTL (Doganlar et al., 2002; Gramazio et al., 2014) and therefore prickliness should be easily removed in backcross generations. Although for fruit size traits negative heterosis was generally observed in the interspecific hybrids, indicating a greater similarity to the wild species, interspecific hybrids with primary genepool species *S. insanum* presented values close to zero, similarly to intraspecific hybrids of eggplant (Rodríguez-Burruezo et al., 2008), indicating intermediate dominance and values intermediate between both parental species. However, hybrids with wild species from the secondary genepool displayed highly negative heterosis, in some cases close to 100% like in interspecific hybrids with *S. anguivi* and *S. tomentosum*, suggesting that in these materials it may be more difficult to recover fruit size in the backcross generations.

In conclusion, the characterization with conventional descriptors and the Tomato Analyzer phenomics tool has allowed a detailed characterization of eggplant, close wild relatives and their interspecific hybrids. The high variation among wild species identified sources of variation and most promising species for traits of interest for eggplant breeding. The fact that interspecific hybrids with primary genepool species *S. insanum* are intermediate or close to eggplant for many traits, may facilitate the use of this species in introgression breeding and supports previous evidence that this species is the ancestor of cultivated eggplant. Also, the high vigor of most interspecific hybrids may be directly exploited by using them as rootstocks. The information obtained here on phenotypic characteristics and heterosis of wild species and interspecific hybrids is of interest for eggplant breeding. Given the adaptation of many wild species to stressful conditions, their utilization in eggplant breeding may result in the development of a new generation of cultivars adapted to climate change challenges.

## Author contributions

JP, SV, PG, and MP conceived and designed the research; PK and MP performed the phenotypic and phenomics characterization; PK, JP, and PG analyzed the data; JP, SV, PG, and MP wrote the manuscript. All authors read and approved the manuscript.

## Funding

This work was undertaken as part of the initiative “Adapting Agriculture to Climate Change: Collecting, Protecting and Preparing Crop Wild Relatives” which is supported by the Government of Norway. The project is managed by the Global Crop Diversity Trust with the Millennium Seed Bank of the Royal Botanic Gardens, Kew and implemented in partnership with national and international gene banks and plant breeding institutes around the world. For further information see the project website: http://www.cwrdiversity.org/. This work has also been funded in part by European Union's Horizon 2020 research and innovation programme under grant agreement No 677379 (G2P-SOL) and from Spanish Ministerio de Economía y Competitividad and FEDER (grant AGL2015-64755-R). Prashant Kaushik is grateful to ICAR for a pre-doctoral grant. Pietro Gramazio is grateful to Universitat Politècnica de València for a pre-doctoral (Programa FPI de la UPV-Subprograma 1/2013 call) contract.

### Conflict of interest statement

The authors declare that the research was conducted in the absence of any commercial or financial relationships that could be construed as a potential conflict of interest.
